# Using MALDI-TOF Mass Spectrometry to Identify Drug Resistant Staphylococcal Isolates from Nonhospital Environments in Brunei Darussalam

**DOI:** 10.1155/2016/8685602

**Published:** 2016-04-03

**Authors:** Ko S. Chong, Siti A. Shazali, Zhen Xu, Ronald R. Cutler, Adi Idris

**Affiliations:** ^1^PAPRSB Institute of Health Sciences, Universiti Brunei Darussalam, Gadong BE1410, Brunei Darussalam; ^2^School of Biological and Chemical Sciences, Queen Mary University of London, London E1 4NS, UK

## Abstract

Drug resistant bacteria have been a growing threat to the community and hospitals due to the misuse of antibiotics by humans, industrialization, and lack of novel antimicrobials currently available. Little is known about the prevalence of drug resistant bacteria in nonhealthcare environments in Brunei Darussalam and about how antibiotic resistant genes are transferred within these environments. Human contact points from different types of environments in Brunei Darussalam, varying from urban to jungle settings, were swabbed and cultured onto selective media to isolate staphylococci bacteria before performing antimicrobial susceptibility testing on the isolates. The identity of the isolates was determined using MALDI-TOF mass spectrometry (MS). Staphylococci isolates resistant to oxacillin were further tested for their minimum inhibitory concentration (MIC). PCR analysis of the* mecA* gene, a gene that confers resistance to oxacillin, is done to determine the level of resistance to oxacillin. Ten different staphylococcal species were identified by MALDI-TOF-MS analysis. Out of the 36 staphylococci isolates, 24 were resistant to multiple antibiotics including two isolates which were oxacillin resistant. Some staphylococci isolates had similar antibiotic resistance profiles to other staphylococci isolates of different species in the same location. This work provides the first-ever evidence of drug resistant staphylococci in the nonhospital environment in Brunei Darussalam.

## 1. Introduction

Antibiotic resistance in bacteria is a growing problem in both developed and developing countries. Since the 1950s mankind has relied on a wide range of antibiotics to treat infections and to protect patients from postsurgical infections. Recently, however, the massive growth in antibiotic resistance in bacterial pathogens has been recognized by the World Health Organization (WHO) and other global healthcare organizations as one of the major problems to healthcare throughout the world. The abuse of over-the-counter antibiotics, international travel, lack of novel antimicrobials, and aging populations in many industrialized countries add to the problem. Despite the known effectiveness of controlling antibiotic use in healthcare settings, drug resistance in the community still remains an issue and little is known as to the spread and transfer of drug resistance among bacteria outside of healthcare settings. “Bacterial resistomes” are communities of bacteria often localised in specific areas and within these environments drug resistance determinants may be freely transferred [1–4]. These environments could be the source of antibiotic resistant bacteria where a collection of antibiotic resistance genes in pathogenic and nonpathogenic bacteria could form a resistome containing a “nexus of genetic diversity” [5, 6].

Staphylococci are recognized as a species of bacteria often closely related to human and animal hosts. The most well-studied member of this genus is methicillin resistant* Staphylococcus aureus* (MRSA), a common hospital and environmental pathogen. However, it is now known that other species within this genus can be involved in the carriage and spread of antibiotic resistance, in both humans and animals [7, 8]. To date, there has been no documented study investigating, in detail, the presence of drug resistant bacteria inside environmental sources in Brunei Darussalam, let alone drug resistant staphylococci. There have been only a handful of studies investigating the incidence of multiple drug resistant staphylococci from nonhealthcare environments including hotels [9], restroom surfaces [10, 11], public transport places [12–16], water parks [17], and fitness centres [18–20]. In this study we evaluated drug resistance in multiple species of staphylococci isolated from a variety of different environmental sources in the Brunei-Muara district of Brunei Darussalam.

## 2. Methodology

### 2.1. Sample Collection

Dry sterile cotton swabs (Omnicell, Mountain View, CA, USA) were used to collect samples from 4 (A, B, C, and D) selected public locations (nonhealthcare) in the Brunei-Muara district, Brunei Darussalam. Approximately 4 cm^2^ areas will be sampled using dry cotton swab and stored in sterile 15 mL tubes (Biologix, Shandong, China). Sampling was carried out on potential human contact points in public locations including bushwalking national parks (Location A), urban recreational parks (Location B), public buses (Location C), and beaches (Location D) over a period of 24 weeks, avoiding rainy days. A total of 102 surface samples were collected from various sites including public exercise equipment, hand rails, park benches, and park ropes. All specimens were transferred to the laboratory within 1–3 hours of the sample being taken.

### 2.2. Culture and Primary Bacteria Isolation

In the laboratory, the swabs were plated onto Nutrient Agar (Nutrient Agar, Oxoid, Basingstoke, UK) medium before swabbing on Mannitol Salt Agar (MSA, Oxoid, Basingstoke, UK) selective medium to select for Gram-positive cocci. Plates were incubated for up to 5 days at 37°C [21]. Bacterial colonies growing on MSA are then subcultured onto another MSA plate to ensure purity of the colonies.

### 2.3. Preparation of Bacterial Isolates for MALDI-TOF-MS Analysis

Colonies (3–5) of overnight cultures were suspended in 300 *μ*L distilled water. The suspension was mixed with 90% absolute ethanol and centrifuged for 2 min at 13000 ×g. The pellets were resuspended in 25 *μ*L of 70% formic acid and then 25 *μ*L pure acetonitrile was added. After mixing, solutions were centrifuged at 13000 ×g for 2 min. One mL aliquots of the supernatant were spotted in duplicate onto MALDI ground steel targets and air-dried for 5 min at room temperature and each target spot was then overlaid with 1 *μ*L a-cyano-4-hydroxycinnamic (HCCA) matrix solution.

### 2.4. Identification of Bacterial Isolates Using MALDI-TOF-MS

All isolates were purified and analysed by matrix-assisted laser desorption/ionization time of flight mass spectrometry (MALDI-TOF-MS) (Microflex LT, MALDI-TOF-MS, Bruker Daltonics, Coventry, UK) in a positive linear mode (2000 to 20000 *m*/*z* range). The resulting spectra for each culture were analysed by MALDI-Biotyper 2.0 software (Bruker Daltonics, Coventry, UK). The software evaluates each spectrum compared to a reference spectrum in the Bruker Taxonomy Database identifying the best match from database records. Results were expressed as scores (QI) from 0 to 3, as recommended by the manufacturer. Scores QI ≤ 1.7 were not considered as reliable identification. A score of QI ≥ 1.7 corresponded to “genus” identification. Only scores higher than QI ≥ 2 were considered a reliable identification of species.

### 2.5. Antimicrobial Susceptibility Testing

Strains were screened for resistance to antibiotics by agar disk diffusion on Iso-Sensitest media (Iso-Sensitest Agar, Oxoid, Basingstoke, UK). Zones of inhibition were evaluated using Mastring M13 and Mastring M48 according to manufacturers instructions (Mast Diagnostics, Merseyside, UK). The minimum inhibitory concentrations (MICs) to oxacillin were additionally evaluated using “M.I.C. evaluators,” antimicrobial gradient strips designed for accurate minimum inhibitory concentration (MIC) values (Oxoid Ltd., Basingstoke, UK). The categories susceptible, intermediate resistant, or resistant were assigned on the basis of the CLSI antimicrobial susceptibility testing standards.

### 2.6. PCR Amplification

Genomic DNA of the isolates was prepared using the commercial MagaZorb® DNA Mini-Prep Kit (Promega, Wisconsin, USA). The* SCCmec* type was determined by detecting* mecA* gene using primers described previously [21]. PCR thermal cycling conditions were 2 min at 94°C, 35 cycles for 30 sec at 94°C, 30 s for 59°C, and 1 min for 72°C. The 100 bp DNA ladder (Promega, Wisconsin, USA) was used as molecular size markers.

## 3. Results and Discussion

The current understanding as to the variety of bacterial species which can exist in microbiomes is still limited [22]. Furthermore the “interactome” between the nonhospital environment and bacteria is not well understood, let alone in Brunei Darussalam. Nonhospital environments, especially with their continual influx of bacterial flora from man, could be the ideal environment for the collection of bacterial resistomes, defined as a collection of antibiotic resistance genes in pathogenic and nonpathogenic bacteria [1, 5, 6, 23].

80 out of the 102 environmental isolates (78.4%) were identified using MALDI-TOF-MS (Table 1). The remaining 23 isolates (22.5%) failed to give a reliable identification. The rates of MALDI-TOF identification at the species level with a score of QI ≥ 2 were 57.5% (46/80) and at genus level with a score of 1.7 ≤ QI ≤ 2 were 42.5% (34/80). In this study, a large number of staphylococci were recovered from our environmental samples. Overall, we identified 36 staphylococcal isolates belonging to 10 species. This included* Staphylococcus aureus* (*n* = 2),* Staphylococcus warneri* (*n* = 2),* Staphylococcus saprophyticus* (*n* = 5),* Staphylococcus hominis* (*n* = 11),* Staphylococcus haemolyticus* (*n* = 5),* Staphylococcus capitis* (*n* = 3),* Staphylococcus epidermidis* (*n* = 3),* Staphylococcus cohnii* (*n* = 3),* Staphylococcus arlettae* (*n* = 1), and* Staphylococcus pasteuri* (*n* = 1). We also identified several other bacteria such as* Bacillus*,* Micrococcus*,* Corynebacterium*,* Kocuria*,* Brevibacterium*,* Kytococcus*, and* Brachybacterium*. Our findings highlight the complexity of the number of bacterial types that can be found on human contact surfaces in the nonhospital environment. Commensal skin bacteria, usually shed from human skin such as* Corynebacterium* and staphylococci, are commonly found in public places such as gyms [18] and restrooms [21]. Results of this study indicate that a wide range of bacterial species were isolated from the nonhospital environment but staphylococci were the most common. In light of this finding, these nonhospital isolates of staphylococci were selected to assess their antibiotic resistance profiles.

Over half of staphylococci isolated in our study carried antibiotic determinants. Staphylococci were isolated from 4 separate locations often on different days, with varying degrees of proximity to the jungle. 25 out of our 36 staphylococci isolates (69.4%) were antibiotic resistant, including resistance to non-*β* lactam antibiotics such as fusidic acid, chloramphenicol, clindamycin, and erythromycin (Table 2). The isolated staphylococci isolates were also resistant to other antibiotics such as gentamicin, ciprofloxacin, streptomycin, and tetracycline. Nine of the isolates were resistant to five or more antibiotics, out of which two isolates (2 A and 6 A) were resistant to oxacillin. In some cases, there were three staphylococcal species (2 A, 4 A, and 7 A) with closely related antibiograms (five of the same class of antibiotic resistance determinants including oxacillin) isolated from different sampling sites in the same location (Table 2). Out of these three staphylococcal species, two were of different staphylococcal species (*S. haemolyticus* and* S. warneri*) (Table 2). This illustrates a possible example of the transfer of drug resistant determinants occurring between different staphylococcal species in the same location. Both* S. haemolyticus* and* S. epidermis* are common coagulase-negative staphylococci (CoNS) found on human skin. CoNS present a potentially significant problem in the healthcare environment and one of the most common agent causing nosocomial infections [24, 25]. It is possible that these two staphylococcal species came from the same human source and transferred to different sites within the same location. Alternatively, the two staphylococcal species could come from a different human source. Examples of similarities of antibiotic resistance determinants in different locations were also detected. For example, antibiograms similar to* S. hominis* (8 A) (4 of the same class of antibiotic resistance determinants) were found in other staphylococcal species from different locations, such as* S. arlettae* (12 B) and* S. hominis* (19 C), potentially indicating a widespread dissemination of these resistance determinants through different staphylococcal species and environments. However, a more thorough investigation of these strains regarding the resistance genes present is warranted to conclusively support this claim. Methicillin resistance is commonly associated with the carriage of the* mecA* gene, which encodes penicillin binding protein PBP2a. Our results also showed that two of the staphylococci environmental isolates were resistant to oxacillin with varying minimum inhibitory concentrations (MICs) (Table 2). PCR analysis showed that these isolates carried the* mecA* gene (Table 2).

To date, there is no documented study investigating the presence of drug resistant staphylococcal species inside major hospitals in Brunei Darussalam. More importantly, the carriage of drug resistant staphylococci outside hospitals is virtually unknown. Existing studies done in Brunei have only investigated human carriage of staphylococci, in both nonhealthcare [26] and healthcare [27] settings. Bacteria not commonly associated with hospital settings can carry resistance determinants. Nonhospital environments such as public transport systems, parks, and gyms are a source of antibiotic resistant staphylococci [10, 12–20]. Our study provides further evidence for environmental “bacterial resistomes” to exist.

## 4. Conclusions

The presence of antibiotic resistance found in staphylococci samples collected from different environments in Brunei Darussalam indicates that some environments may accelerate the spread of antibiotic resistant strains. Attention should be paid to the inspection and control of antibiotic resistant strains in these environments. This work has major implications in the issues of public health and safety in Brunei Darussalam and aims to establish the first-ever epidemiological map of the distribution of drug resistant staphylococci in the country. It will provide comprehensive baseline data for disease control, surveillance, and management at population level. By knowing the type of drug resistant staphylococci circulating in the community, we can devise measures to control the spread of these drug resistant staphylococci.

## Figures and Tables

**Table 1:** Summary of family and genera of bacteria identified by MALDI-TOF-MS.

Family	Genus	Number of isolates
Staphylococcaceae	*Staphylococcus*	36
Bacillaceae	*Bacillus*	9
Micrococcaceae	*Micrococcus*	13
Micrococcaceae	*Kocuria*	8
Corynebacteriaceae	*Corynebacterium*	3
Brevibacteriaceae	*Brevibacterium*	2
Intrasporangiaceae	*Kytococcus*	2
Dermabacteraceae	*Brachybacterium*	7
Total		80

**Table 2:** Resistance profiles and molecular characterisation of antibiotic resistant staphylococci isolated from 24 different sampling spots from 4 locations (A, B, C, and D).

Species	Ss/L	*n*	C	Cl	Cp	E	Fa	G	N	P	O	S	SMX	T	TM	O MIC	*mecA*
*S. epidermis*	1 A	1				R	R			R			R				
*S. haemolyticus*	2 A	1				R	R			R	R		R		R	0.5	+
*S. hominis*	3 A	1				R	R		R				R		R		
*S. saprophyticus*	3 A	1	R				R		R	R			R				
*S. warneri*	4 A	1				R	R			R			R		R		
*S. capitis*	5 A	1	R				R		R	R			R				
*S. epidermis*	6 A	1					R			R	R		R			2	+
*S. haemolyticus*	7 A	1		R		R	R			R			R		R		
*S. hominis*	8 A	1					R			R			R		R		
*S. saprophyticus*	9 A	1					R		R	R			R				
*S. haemolyticus*	10 A	1				R	R						R		R		
*S. saprophyticus*	11 A	1					R		R	R			R				
*S. arlettae*	12 B	1					R			R			R		R		
*S. aureus*	13 B	1								R			R				
*S. capitis*	14 B	1								R							
*S. cohnii*	15 B	2	R			R	R		R	R			R		R		
*S. aureus*	16 B	1								R			R				
*S. capitis*	17 C	1								R			R				
*S. hominis*	18 C	1								R			R		R		
*S. hominis*	19 C	1					R			R			R		R		
*S. hominis*	20 C	1				R	R			R			R		R		
*S. epidermis*	21 D	1								R			R				
*S. pasteuri*	22 D	1								R							
*S. hominis*	23 D	1				R	R						R				
*S. saprophyticus*	24 D	1					R		R	R			R		R		

*n*: similar isolates from each sampling spot but different sites.

Ss/L: sampling spot/location codes A, B, C, and D.

Cp: ciprofloxacin; Cl: clindamycin; C: chloramphenicol; E: erythromycin; Fa: fusidic acid; G: gentamicin; N: novobiocin; O: oxacillin; P: penicillin; SMX: sulfamethoxazole; S: streptomycin; T: tetracycline; TM: trimethoprim.

O MIC: minimum inhibitory concentration to oxacillin (*µ*g/mL).

## References

[1] Perry J. A., Wright G. D. (2014). Forces shaping the antibiotic resistome. *BioEssays*.

[2] Gaze W. H., Krone S. M., Joakim Larsson D. G. (2013). Influence of humans on evolution and mobilization of environmental antibiotic resistome. *Emerging Infectious Diseases*.

[3] Finley R. L., Collignon P., Larsson D. G. J. (2013). The scourge of antibiotic resistance: the important role of the environment. *Clinical Infectious Diseases*.

[4] Olivares J., Bernardini A., Garcia-Leon G., Corona F., Sanchez M. B., Martinez J. L. (2013). The intrinsic resistome of bacterial pathogens. *Frontiers in Microbiology*.

[5] Wright G. D. (2007). The antibiotic resistome: the nexus of chemical and genetic diversity. *Nature Reviews Microbiology*.

[6] Wright G. D. (2010). The antibiotic resistome. *Expert Opinion on Drug Discovery*.

[7] Livermore D. M. (2000). Antibiotic resistance in staphylococci. *International Journal of Antimicrobial Agents*.

[8] Wendlandt S., Feßler A. T., Monecke S., Ehricht R., Schwarz S., Kadlec K. (2013). The diversity of antimicrobial resistance genes among staphylococci of animal origin. *International Journal of Medical Microbiology*.

[9] Xu Z., Mkrtchyan H. V., Cutler R. R. (2015). Antibiotic resistance and mecA characterization of coagulase-negative staphylococci isolated from three hotels in London, UK. *Frontiers in Microbiology*.

[10] Gibbons S. M., Schwartz T., Fouquier J. (2015). Ecological succession and viability of human-associated microbiota on restroom surfaces. *Applied and Environmental Microbiology*.

[11] Mkrtchyan H. V., Xu Z., Cutler R. R. (2015). Diversity of SCCmec elements in Staphylococci isolated from public washrooms. *BMC Microbiology*.

[12] Zhou F., Wang Y. (2013). Characteristics of antibiotic resistance of airborne Staphylococcus isolated from metro stations. *International Journal of Environmental Research and Public Health*.

[13] Yeh P. J., Simon D. M., Millar J. A., Alexander H. F., Franklin D. (2011). A diversity of Antibiotic-resistant *Staphylococcus spp.* in a Public Transportation System. *Osong Public Health and Research Perspectives*.

[14] Otter J. A., French G. L. (2009). Bacterial contamination on touch surfaces in the public transport system and in public areas of a hospital in London. *Letters in Applied Microbiology*.

[15] Simões R. R., Aires-de-Sousa M., Conceição T., Antunes F., da Costa P. M., de Lencastre H. (2011). High prevalence of EMRSA-15 in Portuguese public buses: a worrisome finding. *PLoS ONE*.

[16] Stepanović S., Ćirković I., Djukić S., Vuković D., Švabić-Vlahović M. (2008). Public transport as a reservoir of methicillin-resistant staphylococci. *Letters in Applied Microbiology*.

[17] Davis T. L., Standridge J. H., Degnan A. J. (2009). Bacteriological analysis of indoor and outdoor water parks in Wisconsin. *Journal of Water and Health*.

[18] Mukherjee N., Dowd S. E., Wise A., Kedia S., Vohra V., Banerjee P. (2014). Diversity of bacterial communities of fitness center surfaces in a U.S. metropolitan area. *International Journal of Environmental Research and Public Health*.

[19] Markley J. D., Edmond M. B., Major Y., Bearman G., Stevens M. P. (2012). Are gym surfaces reservoirs for *Staphylococcus aureus*? A point prevalence survey. *American Journal of Infection Control*.

[20] Ryan K. A., Ifantides C., Bucciarelli C. (2011). Are gymnasium equipment surfaces a source of staphylococcal infections in the community?. *American Journal of Infection Control*.

[21] Mkrtchyan H. V., Russell C. A., Wang N., Cutler R. R. (2013). Could public restrooms be an environment for bacterial resistomes?. *PLoS ONE*.

[22] Turnbaugh P. J., Ley R. E., Hamady M., Fraser-Liggett C. M., Knight R., Gordon J. I. (2007). The human microbiome project. *Nature*.

[23] Perry J. A., Westman E. L., Wright G. D. (2014). The antibiotic resistome: what's new?. *Current Opinion in Microbiology*.

[24] Edern A., Fines-Guyon M., Castrale C., Ficheux M., Ryckelynck J.-P., Lobbedez T. (2012). Ecology and mechanisms of bacterial resistance to antibiotics in peritonitis. *Nephrologie & Therapeutique*.

[25] Koksal F., Yasar H., Samasti M. (2009). Antibiotic resistance patterns of coagulase-negative staphylococcus strains isolated from blood cultures of septicemic patients in Turkey. *Microbiological Research*.

[26] Abdul Malik N. F., Muharram S. H., Abiola O. (2014). *Staphylococcus aureus* nasal carriage in young healthy adults in Brunei Darussalam. *Brunei International Medical Journal*.

[27] Abiola O. (2014). *Staphylococcus aureus* antibiotic resistant patterns altered in student nurses after clinical experience. *Asian Journal of Biomedical and Pharmaceutical Sciences*.

